# Indebtedness and mental health in China: the moderating roles of income and social support

**DOI:** 10.3389/fpubh.2023.1279683

**Published:** 2024-01-04

**Authors:** Jiankun Liu, Yueyun Zhang

**Affiliations:** ^1^School of Humanities and Social Sciences, Harbin Engineering University, Harbin, China; ^2^School of Social Sciences, Harbin Institute of Technology, Harbin, China

**Keywords:** indebtedness, mental health, income, social support, China

## Abstract

**Objective:**

To explore the effect of indebtedness on mental health and the moderating effects of two types of coping resources (i.e., income and social support) in the Chinese context.

**Methods:**

41,274 adults from four waves of China Family Panel Studies conducted in 2012, 2016, 2018, and 2020. Center for Epidemiologic Studies Depression Scale was used for investigation. Pooled ordinary least squares regressions were used to examine the effect of indebtedness on mental health and the moderating effects of income and social support. Stata 16.0 was used to conduct data analysis.

**Results:**

The results showed that indebtedness had an adverse effect on mental health among Chinese adults. Furthermore, debtors with higher incomes showed fewer mental disorders than those with lower incomes. In terms of social support, monetary support from relatives was able to moderate the negative effects of indebtedness; however, the moderating effects of emotional support were negligible.

**Conclusion:**

The results of this study indicated the adverse mental health outcomes of indebtedness in emerging economies and highlighted that economic resources played protective roles against debtors’ mental disorders.

## 1 Introduction

Mental disorders, such as depression and anxiety, not only contribute to physical illnesses, decreased quality of life, and increased mortality rate (1), but also produce immense economic burdens for individuals, families, and societies (2). Following previous research that examined health consequences using the stress process model, mental disorders have been conceptualized as an outcome of stressors (3). Economic hardship, as a money-related stressor, has been well documented to be the risk factor resulting in severe mental disorders (4, 5). Particularly, with the expansion of the credit market, household debt has boomed among developed and developing countries, thus becoming the key predictor of family economic conditions. Although the role of indebtedness has been subject to growing scholarly interest in the field of health studies (6), findings have been mixed. The reasons for the discrepancies may include differences in the data and samples used in the studies, measurements of indebtedness and mental health, and estimation methods. Studies have indicated that indebtedness is associated with mental disorders, including anger, anxiety, and depression (7–10). Yet, another set of studies found that household debt had a positive link with less depressive symptoms among married couples and improved self-esteem and mastery (11, 12).

Furthermore, although existing studies have examined the link between debt and mental health, they only provided evidence from developed countries. Indeed, the topic is especially important for emerging economies because they not only had a faster growth rate of household debt but also constructed a distinct debt structure compared to developed countries. Particularly, China, as the largest emerging economy, has the largest credit market among emerging economies, and levels of household debt have been similar in size to developed ones like the US (13). Under the situation where the global credit market has undergone a drastic transition, conducting research using the data from emerging economies, such as China, will offer the opportunity to clarify the association of indebtedness with mental health and then generate new information for researchers and policymakers.

Finally, although many studies have documented the negative associations between indebtedness and mental disorders, few have further explored the potential mechanisms that may serve to protect debtors’ mental health. According to the stress-buffering model, access to and use of coping resources can alleviate the detrimental effects of stressors (14). Specifically, Income is the main measure of personal resources, which has been documented to be associated with borrowers’ abilities to repay the debt and further impacts their perception of financial strain (15). However, whether the link between debt and mental health varies across income groups is unclear. Additionally, social support, as coping resources provided by others, has been linked to reduced mental health issues of individuals (16). However, the potential role of social support in the link between stressors and mental disorders was less well investigated, with mixed results (17–19). Indeed, debt, a money-related stressor, reflects accumulated economic adversity (20), but the extent to which debt interacts with social support to affect mental health remains unexplored.

**Figure 1:**
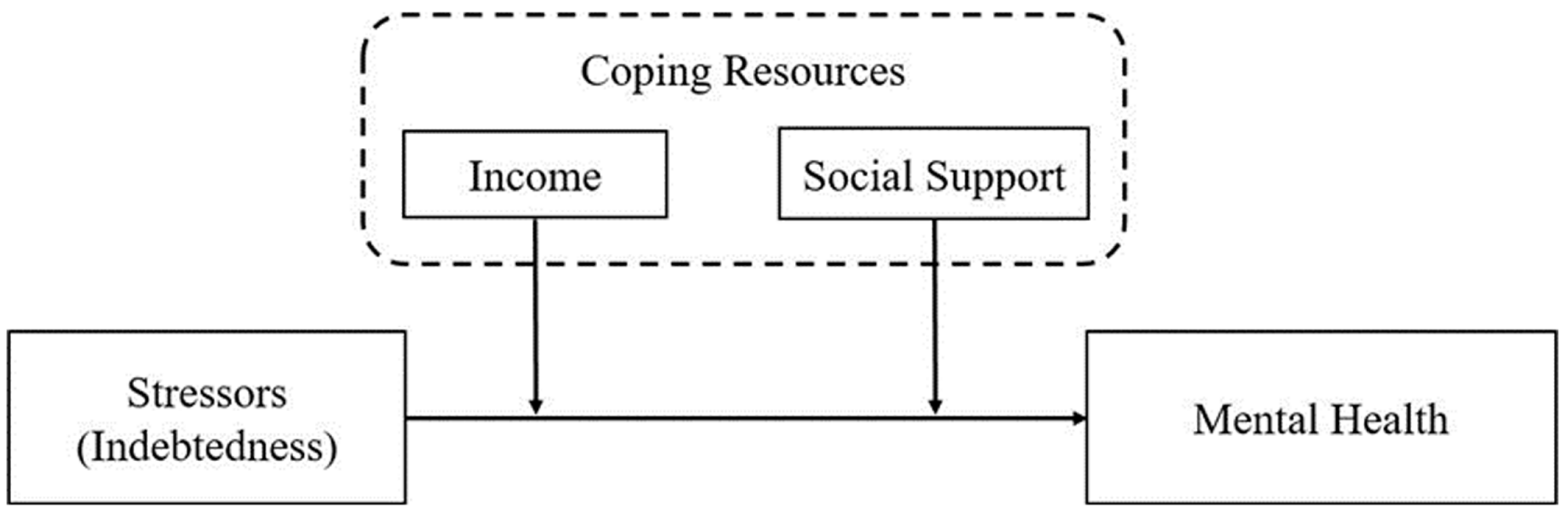
The conceptual model.

Drawing on the stress process and stress-buffering paradigm theoretical framework, the current study conceptualized debt as a money-related stressor and two forms of coping resources (i.e., income and social support) as a buffer against the potential negative effect of indebtedness (see Figure 1). According to this framework, we explored the relationship between indebtedness and mental health among adults in the Chinese context, with a particular focus on the moderating roles of income and social support. At the method level, the analysis was based on a large-scale and national representative sample from China Family Panel Studies (CFPS). This study provided a deeper understanding of the link between indebtedness and mental health and the moderating roles of coping resources. Based on the obtained results, intervention strategies can be developed to protect individuals from the adverse impact of indebtedness on mental health in emerging economies.

## 2 Literature review and hypothesis

### 2.1 Indebtedness and mental health

The stress process model postulates that stressors, primarily referred to as stressful life events and chronic strains, may lead to the emergence and accumulation of mental disorders (3). Generally, socioeconomic status (SES) reflects individuals’ abilities to utilize economic resources. Thus, those with lower SES are more likely to be trapped in unresolved financial hardship, which has detrimental effects on physical and psychological development. A sizeable literature following the stress process paradigm has documented that mental health was tightly associated with SES, mainly measured by education, income, and occupational status (21–23). However, these traditional indicators of SES have been criticized for underestimating the impacts of economic hardship on mental health and generating mixed results (24, 25).

In contrast, debt is a measure of accumulated economic hardship, thus capturing long-term deprivation of economic resources (20). A growing body of literature has focused on the crucial role of debt in the formation of a process of stress and further investigated the association between indebtedness and mental health, but the findings were not inconclusive. One strand of literature, following the stress process model, has indicated that debtors suffered from mental disorders due to unbearable economic pressures (26). This is because Indebted households have to allocate a significant share of existing resources to debt repayment, thus resulting in the deduction of disposable resources (27). in addition, when debt is not completely repaid, debtors will be collected by creditors (e.g., banks and usurers), and thus are often assailed by financial insecurity (28). Previous studies conducted in the US and UK have revealed that unbearable economic burdens due to excessive debt could induce a serious of psychological distress, such as anger, anxiety, depression, etc. (7–10, 29).

Another set of literature held that debt could produce positive mental health outcomes through the material rewards of borrowing money. This literature was based on the need satisfaction theory, which proposes that satisfaction of material needs is generally regarded as fundamental “nutriments” for individual psychological development (30). Therefore, indebted households may have more opportunities to smooth consumption, invest in assets like homeownership, and make major purchases on credit in the present (31), each of which is the source of satisfying material needs. Empirical studies have revealed that debt is an important predictor of greater mental health. For instance, using the data from the National Study of Families and Households (NSFH), Dew found that indebtedness directly resulted in depressive symptoms among married couples in the US, even after controlling for variables in the stress process paradigm (11). With the same data, Dwyer et al. found that both long- and short-term debt increased mastery and self-esteem among young people (12).

The two potential methodological problems in the two strands of literature above may be the source of inconsistent findings. First, many studies have only utilized self-reported measures of financial strains due to indebtedness, such as worries about debt or concerns about never being able to repay debt (7, 8). However, the respondents’ subjective perceptions of economic hardship are more likely to deviate from reality in an interview setting (32). A recent study has found that a measure of self-perceived debt stress had a stronger effect on individuals’ mental health than an objective measure of indebtedness (33). Second, the results estimated in prior studies may be biased due to the endogeneity in linear regression models and sample selection bias, thus posing formidable challenges to identifying the exact relationship between indebtedness and mental health. Specifically, reverse causality, as the main source of the endogeneity problem, refers to the two-way causation between mental health on the one hand and debt on the other. With regard to the sample selection bias, because both debt and mental health may be influenced by some individual and household characteristics, the correlations between the two are spurious. The current study attempted to address those methodological problems by estimating the effect of objective indicators of indebtedness on self-reported mental disorders using multiple causal inference methods.

Furthermore, the link between indebtedness and mental health may be different in a variety of institutional and cultural settings. Existing findings in previous research were based on the data from developed countries. Yet, the topic of debt and mental health is increasingly important for emerging economies because they are undergoing a rapid transition of financial institutions observed in developed ones, but borrowing behaviors are still constrained by traditional values. For instance, due to the explosion of housing prices and the rapid popularity of financial products, China has the largest credit market among emerging economies. As of 2019, household debt in China has surged to 56% of GDP, which was similar in size to the US level before the 2008 financial crisis, and even credit card debt in China has recently exceeded the US level in absolute terms (13). However, borrowing money is not encouraged in the Chinese value system (34), which is reflected in some traditional proverbs, such as “wu zhai yi shen qing (One only feels free when he or she has no debt).” Consequently, the Chinese have less preference for risk and make savings a priority, thus leading to the continuous increase in the household saving rate in the last three decades (35). Driven by the initiative of risk aversion, households with higher levels of debt are likely to have an elevated exposure to financial strains, and such exposure may be the source of mental disorders. Based on the above discussion, we proposed the following hypothesis:

*Hypothesis 1*: Indebtedness is negatively associated with mental health among adults in China.

### 2.2 The potential moderators of income and social supports

Although indebtedness would be expected to lead to negative mental health outcomes through economic stress following the stress process paradigm, debtors may not all equally suffer from mental disorders to the same extent. This difference may result from the protective roles of coping resources, which can buffer the adverse effects of stressors following the stress-buffering model. The study investigated the potential moderating roles of two types of coping resources, i.e., income and social support.

#### 2.2.1 Income

Income, as the key dimension of SES, is the most important component of individuals’ and households’ economic resources controlled by themselves (36). Generally, income will accumulate over time without violent shocks due to environmental changes, such as unemployment, natural disasters, and economic crises, thus providing continuous financial support for all family members. As a result, income has a sustained impact that works as a buffer against stressful life events, especially financial strains, and thus plays a critical and central role in individuals’ physical and psychological development (37). The abundance of literature conducted in developed countries has indicated that higher income levels are significantly associated with better mental health outcomes. Specifically, individuals in economically advantaged households had lower levels of depression and greater amounts of happiness than those in households with fewer economic resources (21). Another set of studies using the data from emerging economies provided evidence for the link between income and mental health. Based on a sample of Hubei province in China, Li et al. found that participants suffering heavy income losses had more mental disorders during the COVID-19 pandemic (38).

Moreover, income may be a moderator of mental disorders among debtors. Generally, only when borrowers have the ability to repay debt can indebtedness produce material and well-being benefits (31). Thus, an imbalance between the usage of debt and the resources available to debtors will undermine their mental health, and in turn, sufficient resources may mitigate the negative effect of indebtedness. The stress-buffering model holds that economic resources like income are critical protective factors because they can help individuals overcome hardship and further promote physical and psychological development (39). Indeed, income not only provides access to debt but also affects the repayment ability, which is associated with whether debtors suffer from mental disorders. However, only a few studies have focused on the buffering effect of income. For instance, using the British Household Panel Survey (BHPS), Brown et al. found a negative association between indebtedness and psychological well-being and anticipated that the adverse effect could be eliminated when debtors obtain a 7% increase in their monthly income (40). However, they did not provide empirical evidence for the estimation.

To sum up, despite the rapid growth of literature concerning the link between income and mental health, the protective role of income remains unclear, especially in emerging economies. To address this research gap, this study aimed to examine whether income attenuates the adverse effect of indebtedness on mental health. Based on the discussion above, we suggested that the ability to repay debt was different for indebted households with different income levels, and high income can produce a buffering effect on adverse mental health outcomes of indebtedness. The specific hypothesis was as follows:

*Hypothesis 2*: High levels of income have a mitigating effect on the negative association of indebtedness and mental health.

#### 2.2.2 Social support

Social support has been defined as perceived, instrumental, and expressive support provided by partners interacting with recipients, including family members, friends, and communal members (41). The main effect model, proposed to explain the direct association between social support and mental health, holds that individuals who have perceived or received social support would show better mental health in all living environments (42). There is a plethora of evidence on the positive relationship between social support and mental health. A longitudinal study using the General Social Survey (GSS) has revealed that the decrease in social support from neighbors was the major source of the decline in reported happiness among Americans over a 30-year period (43). Another research using multiple sources of data covering 49 countries found that when maintaining intimate relationships with family members or positively engaging in social activities, individuals received greater economic and emotional support, which had a positive effect on happiness and life satisfaction, regardless of countries’ development level (44). A direct link of social support with mental health has also been found in China. For instance, two studies based on the samples of Chinese adolescents and older adults found that those who received support from their relatives and friends were less likely to suffer from psychological illnesses (16, 45).

Furthermore, the stress-buffering model suggests that the positive mental health outcomes of social support may be more evident when one is in a state of high stress (42). This is because individuals will be more effective in mobilizing material and psychological resources in the presence of adequate support, thus relieving the negative feelings caused by stressful life events (4). Therefore, social support has been conceptualized as an important protective factor that can alleviate the adverse effects of stressors on mental health, yet previous research provided mixed findings. On the one hand, several studies have revealed that social support could mitigate mental disorders among individuals beset by stressful life events. Aslund et al. found using the Sweden data that individuals with high financial stress and high tangible social support had higher psychological well-being and fewer psychosomatic symptoms (17). Among Chinese older adults, Yu and Liu found that support from family members could moderate the positive association between stressful life events and depressive symptoms (18). Other studies did not confirm the buffering effect of social support on the link between stressors and mental health. Szkody and McKinney found that neither perceived nor received social support significantly moderated the relationship between traumatic events (e.g., experiencing natural disasters, life-threatening accidents, and death of a loved one) and depression among American adolescents when variables about parental depressive issues were added to models (19).

Overall, previous research has made earnest efforts to investigate whether social support serves as a buffer between stressors and mental health, but specific conclusions were still controversial and inconsistent. Moreover, debt, a money-related stressor, was not considered in the existing literature. Thus, it remains unclear whether social support has a buffering effect on the association between indebtedness and mental health. To bridge these gaps, the current study examined whether social support alleviated the adverse mental health outcomes of indebtedness in the Chinese context. Given the mixed moderating role of social support in prior studies, we evaluated the following hypotheses:

*Hypothesis 3a*: High levels of social support have a mitigating effect on the negative association of indebtedness and mental health.

*Hypothesis 3b*: High levels of social support have no mitigating effect on the negative association of indebtedness and mental health.

## 3 Method

### 3.1 Data

The data used in the current study was obtained from four waves (2012, 2016, 2018, and 2020) of CFPS, which was conducted by the Institute of Social Science Survey (ISSS) of Peking University. The baseline CFPS survey was officially launched in 2010, followed by five waves in 2012, 2014, 2016, 2018, and 2020. CFPS uses a multistage probability-proportional- to-size (PPS) sampling method with implicit stratification, wherein administrative districts (provinces, cities, and counties) and socioeconomic status (mainly local gross domestic product [GDP] *per capita*) serve as the main stratification variables, and thus approximately 15,000 households were sampled in each wave of the survey of all age groups in 25 provinces, representing 94.5% of the population in mainland China. Moreover, the data collected in CFPS is divided into two subsets of individuals and families. As a nationally representative longitudinal survey, CFPS provides scholars with the most comprehensive and highest-quality data on contemporary China and has been used in social science research.

The 2012, 2016, 2018, and 2020 waves of CFPS were finally employed in the study because items measuring mental health were identical in the four waves of the survey. The original data for the four waves was collected from 48,552 individuals aged below 18. Following other studies (10), we excluded individuals above 65 to focus on working-age adults. Business owners were also excluded in order to ensure that the measures of indebtedness reflected household rather than corporate debt. These criteria resulted in the exclusion of just over 14% of the initial sample (6,983 observations). During data cleaning, 1,809 observations were dropped because of missing data on mental health, and 3,804 were dropped because of missing data on indebtedness and other control variables. We employed the multiple imputation method to impute data. After matching with the subset of families according to the family ID, a total of 41,274 adults were included in the present study.

### 3.2 Measures

#### 3.2.1 Mental health

The main dependent variable in this study was the mental health status of adults in China. Following existing studies (46), the study used the 8 items of the Center for Epidemiologic Studies Depression Scale (CESD-8) to measure mental health. In CPES, respondents were asked how they had felt and behaved in the past week in terms of specific emotions or behavior (e.g., “I feel struggling to do anything”) on a four-scale metric ranging from “almost never (less than 1 day),” “sometimes (1–2 days),” “often (3–4 days),” “most of the time (5–7 days).” These responses were assigned a value of 0 to 3, respectively. The total CESD-8 scores range from 0 to 24, with higher scores indicating more severe depression.

#### 3.2.2 Indebtedness

The main independent variable was indebtedness. In CPES, adult respondents were asked if their households were indebted, and if so, the approximate amount of total debt was then asked. Following other studies (10), we modeled indebtedness in several ways. The first specification was a dichotomous variable indicating a household had any debt (yes vs. no). The second measure was total debt, which was assessed by the self-reported amount of total debt. Additionally, we further constructed a relative measure of the total debt-to-assets ratio, which allowed us to test the robustness of results using the absolute measures of indebtedness.

#### 3.2.3 Income

In this study, two indicators were used to measure income levels following existing studies (47). The first indicator was income levels (logarithm, continuous), which were measured by household annual income. The second measure was a binary variable equal to 1 if the respondents lived in high-income households and 0 otherwise. Specifically, the high-income group referred to the top 25% of households with respect to the annual income, and the low/middle-income group referred to the second, third, and fourth quartiles of households.

#### 3.2.4 Social support

In this study, received support from others to respondents included monetary and emotional support following prior studies (16). We measured monetary support by the value of economic help that the respondents had received from their relatives and friends during the year prior to when the survey was carried out. Emotional support was indicated by parent–child relationship quality and couple relationship quality following previous research (48). Parent–child relationship quality was measured by respondents’ self-rated emotional closeness with their children, and the measurement item was “What was the closeness of the relationship with your children during the past 6 months?” Couple relationship quality was measured by respondents’ satisfaction with their spouses, and the measurement item was “How satisfied are you with your relationship with your spouses?” Responses to the two questions ranked on a 5-point scale, with 1 standing for not close at all or not at all satisfied, 5 very close or completely satisfied. It is worth noting that these items measuring social support existed in the 2018 wave of CFPS. Therefore, we tested the moderating roles of social support with the analysis of CPES2018.

#### 3.2.5 Covariates

This study controlled for several sociodemographic, socioeconomic, and health conditions characteristics potentially associated with depressive symptoms following previous studies (47). These variables consisted of gender (male vs. female), age (continuous), marital status (married vs. not married), Hukou status (urban vs. rural), educational level (illiterate, elementary school, junior high school, senior high school, and bachelor’s degree or higher), employment status (employed vs. unemployed), diagnosed with chronic diseases (yes vs. no), the number of family members (continuous), and total assets (logarithm, continuous).

### 3.3 Statistic analysis

Given that the data came from four waves of CFPS, pooled ordinary least squares (POLS) regression models were used to examine the relationship between indebtedness and mental health and the moderating roles of income and social support. Robust standard errors were clustered at the county level to account for the correlations between observations within a county. The general structure of the model was specified as:

$$Y_{\mathrm{it}}=f\left(\beta_{1}D_{\mathrm{it}}+\beta_{2}M_{\mathrm{it}}+\beta_{3}D_{\mathrm{it}}M_{\mathrm{it}}+\beta_{4}X_{\mathrm{it}}+\varepsilon_{\mathrm{it}}\right)\#$$

in which *Y* represents the measures of mental health, *D* denotes three measures of indebtedness, and *M* indicates the measures of income and social support. *X* is a vector of the control variables. ε is the error term. The coefficient $\beta_{1}$ captures the relationship between indebtedness and adults’ mental health, and the coefficient vector $\beta_{3}$ indicates the moderating effects of income and social support. The year dummies are added to control for omitted time-related characteristics. We estimated two specifications of this model. The first included only the measures of indebtedness and control variables. The moderating term ( $D_{\mathrm{it}}M_{\mathrm{it}}$ ) was added one at a time to the regression in the second specification.

The advantage of POLS regressions is that it could capture the between-unit variations and include year dummies that could control for the unobserved between-unit characteristics. Yet, POLS has one limitation, i.e., it can not control for the unobserved stable characteristics that differ between debtors and non-debtors, resulting in biased estimation results. An alternative approach to address this issue is using a fixed-effects (FE) model for four-period panel data, which can adjust for time-invariant unobservable characteristics by including individual fixed effects (47). Due to space limitations, we presented the results of POLS in the next section, and the FE model results are included in the Supplementary material.

Finally, given the potential endogeneity problem in baseline models and sample selection bias, we checked the robustness of baseline estimation results by using the instrumental variable (IV) method and propensity score matching (PSM) method, thus identifying the causal effect of indebtedness on mental health. The software Stata 16.0 was utilized for statistical analysis.

## 4 Results

### 4.1 Descriptive statistics

Table 1 presented the characteristics of the sample in all four waves of CFPS. The average CESD-8 scores of Chinese adults were 5.28. On average, over 35% of Chinese households were in debt, and the average amount of total debt was 4.93 ten thousand yuan (RMB). The mean ratio of total debt to assets was 0.16.

**Table 1:** Descriptive statistics of the sample.

Variables	Obs.	Mean	Std. dev.	Min	Max
Mental health					
CESD-8 scores	41,274	5.28	3.83	0	24
Indebtedness					
Any debt	41,274	0.36	0.48	0	1
Total debt	41,274	4.93	18.31	0	1,015
Total debt to assets	41,274	0.16	0.48	0	5.00
Moderators					
Annual income	41,274	8.26	14.14	0	915.88
High-income group	41,274	0.26	0.44	0	1
Monetary support from relatives	41,274	0.12	1.20	0	100
Monetary support from friends	41,274	0.02	0.25	0	15
Parent–child relationship quality	41,274	4.31	0.76	1	5
Couple relationship quality	41,274	4.49	0.85	1	5
Covariates					
Male	41,274	0.49	0.50	0	1
Age	41,274	46.35	11.28	18	65
Married	41,274	0.98	0.15	0	1
Urban Hukou	41,274	0.24	0.43	0	1
Education level	41,274	2.65	1.21	1	5
Employed	41,274	0.88	0.33	0	1
Any chronic diseases	41,274	4.47	1.92	1	2
Number of family members	41,274	0.15	0.36	0	1
Total assets	41,274	63.84	140.64	0	6,180

M, mean; SD, standard deviation.

The mean annual income of surveyed households was 8.26 ten thousand yuan (RMB), and approximately one-quarters of respondents lived in high-income households. On average, the values of monetary support from respondents’ relatives and friends were 0.12 and 0.02 ten thousand yuan (RMB), respectively. The mean scores of parent–child relationship quality and couple relationship quality were 4.31 and 4.49, respectively, indicating that most respondents had closer relationships with their children and spouses.

### 4.2 Indebtedness and mental health

#### 4.2.1 Baseline results

Table 2 presented results from POLS regressions regarding the association between six indicators of indebtedness and CESD-8 scores. The estimate from Model 1, which included a binary indicator for holding any debt as the key predictor and controlled for a set of covariates, revealed that holding any debt was associated with higher CESD-8 scores, and this association was statistically significant (*p* < 0.001), holding other characteristics constant. Consistent with the results for holding any debt (Model 1), we found in Model 2 that the amount of total debt (logarithm) was also positively associated with CESD-8 scores, and this estimate attained statistical significance (*p* < 0.001), suggesting that indebtedness was the contributor of mental disorders. Moreover, we replaced the absolute amount of debt with the relative amount of debt (to assets). The results in Model 3 of Table 2 showed the ratio of total debt to assets was positively associated with CESD-8 scores (*p* < 0.001). The results in Table 2 were confirmed by the fixed-effects results (see Supplementary Table S1), regardless of measures of indebtedness. These results supported Hypotheses 1.

**Table 2:** POLS regressions on the association of indebtedness with mental health.

Variables	Model 1	Model 2	Model 3
	DV: CESD-8 scores		
Any debt	0.736^***^		
	(0.055)		
Total debt (logarithm)		0.289^***^	
		(0.025)	
Total debt to assets			0.449^***^
			(0.049)
Male	−0.714^***^	−0.707^***^	−0.700^***^
	(0.054)	(0.054)	(0.054)
Age	−0.004	−0.004	−0.006^+^
	(0.003)	(0.003)	(0.003)
Married	−0.509^***^	−0.511^***^	−0.493^***^
	(0.122)	(0.123)	(0.124)
Urban hukou	−0.274^**^	−0.285^**^	−0.305^***^
	(0.088)	(0.089)	(0.089)
Education level (ref.: Illiterate)			
Elementary school	−0.600^***^	−0.604^***^	−0.618^***^
	(0.102)	(0.102)	(0.102)
Junior high school	−0.901^***^	−0.907^***^	−0.931^***^
	(0.104)	(0.104)	(0.104)
Senior high school	−1.025^***^	−1.043^***^	−1.062^***^
	(0.109)	(0.109)	(0.110)
Bachelor’s degree or higher	−0.854^***^	−0.889^***^	−0.862^***^
	(0.140)	(0.141)	(0.142)
Employed	−0.135	−0.133	−0.120
	(0.084)	(0.084)	(0.085)
Any chronic diseases	1.686^***^	1.691^***^	1.705^***^
	(0.075)	(0.076)	(0.077)
Number of family members	0.023	0.028	0.031
	(0.020)	(0.020)	(0.020)
Annual income (logarithm)	−0.331^***^	−0.358^***^	−0.325^***^
	(0.044)	(0.044)	(0.044)
Total assets (logarithm)	−0.307^***^	−0.342^***^	−0.276^***^
	(0.033)	(0.033)	(0.032)
Constant	7.800^***^	8.072^***^	7.947^***^
	(0.229)	(0.229)	(0.228)

^+^*p* < 0.1, ^*^p < 0.05, ^**^p < 0.01, ^***^p < 0.001. Robust standard errors in parentheses.

#### 4.2.2 Robustness check using The IV method

The results using PLOS regressions may be biased due to the endogeneity problem, mainly arising from reverse causality. Specifically, individuals’ mental health may be influenced by the pressure due to indebtedness, and in turn, may affect their choices of borrowing, which is directly related to debt levels (49). To control possible endogeneity, a two stage instrumental variable approach was performed to identify the causal relationship between household and mental health.

We used the amount of total debt aggregated at the community level as an instrumental variable named *TDCL*, which fulfilled the following essential assumptions for validity for instrumental variables (50). One is the exclusion restriction assumption. Indeed, the average debt levels of the community, as a macroeconomic factor, may not directly affect individuals’ mental health. Individuals’ resources, measured at the cluster level, have long been used as an instrumental variable in the existing literature; for example, the research on the impact of social capital on mental health (51). Thus, the assumption can be fulfilled because individual mental health is not affected by the aggregated household at the community level once single households’ debt is taken into account.

Another one is the relevance assumption, i.e., the average household debt at the cluster level of a community has a strong correlation with the debt levels of a single household. The assumption is feasible at the theoretical level because individuals’ choices in economic activities, such as investment and consumption, have been documented to be affected by neighbors, as the tight geographical relationship makes it possible to exchange information and resources (52). Moreover, we ran OLS regressions of the instrumental variable (*TDCL*) and single households’ debt levels. The results indicated that the coefficients for *TDCL* on three measures of indebtedness were economically and statistically significant (*p* < 0.001) (see Supplementary Table S2), implying a strong and robust positive association between the aggregate of debt at the community level and the probability of holding debt and the amount of debt at the household level.

Given that CESD-8 scores were identified as the dependent variable, we first performed two stage least square (2SLS) regressions (see Supplementary Table S3). The Durbin–Wu–Hausman test indicated that all three models were subject to the endogeneity problem. This means that we needed to perform 2SLS regressions. Moreover, the Wald F statistics were all higher than the general standard of 10, indicating that the instrument variable (*TDCL*) was not a weak instrumental variable. As compared to the endogenous model where measures of indebtedness were not instrumented (see Table 2), the three measures of indebtedness were tightly associated with CESD-8 scores (*p* < 0.001), and magnitudes of the coefficients significantly increased, implying that the endogeneity problem may lead to serious underestimation of the effect of indebtedness on mental health.

#### 4.2.3 Robustness check using the PSM method

To alleviate the sample selection bias, we implemented the PSM to estimate the treatment effect of being indebted, as compared to not being indebted, on mental health. The PSM method can relax the linear assumption for the relationship between indebtedness and mental health, which helps to avoid the selection bias arising from observed control variables. The prerequisite of performing the PSM method is extracting two subgroups from the full sample according to the independent variables as dichotomous measures. Therefore, we used the measure of holding any debt as the treatment variable and divided the full sample into the treatment group (individuals in indebted households) and the control group (individuals in non-indebted households).

In the auxiliary analysis using the PSM method, we used nearest neighbor matching (NM), radius matching (RM), kernel matching (KM), and local linear matching (LM), respectively, for the CESD-8 scores. We employed the same list of control variables as those in the models of Table 2 to predict the propensity score. We reported the average treatment effect on the treated (ATT), imposing the common support restriction to improve the quality of the matches (see Supplementary Table S4). The results showed ATT in all models was highly significant, suggesting that individuals in indebted households had higher mental disorders than those in non-indebted households.

Through a series of benchmark estimations and robustness checks, a robust empirical causal effect was observed, i.e., indebtedness, measured by holding any debt, absolute and relative debt levels, was an important contributor to mental disorders among Chinese adults.

### 4.3 Moderating roles of income and social support

#### 4.3.1 Income

Table 3 showed the moderating role of income in the link between indebtedness and mental health (CESD-8 scores as the dependent variable) using POLS regressions. A significant interaction term of total debt and income was found in Model 1 (*b* = −0.096, *p* < 0.001). Specifically, after controlling for covariates, for every ten thousand yuan (RMB) increase in annual income, CESD-8 scores of indebted adults decreased by 0.096 points. We further investigated the effects of indebtedness on mental health among different income groups. The interaction term of total debt and the high-income group was highly significant (*p* < 0.001), which indicated that among the indebted adults, those who lived in the high-income households had lower levels of depression than those who lived in the low/middle income households.

**Table 3:** POLS regressions on the moderating effects of income.

Variables	Model 1	Model 2
	DV: CESD-8 scores	
Total debt (logarithm)	0.510^***^	0.359^***^
	(0.063)	(0.031)
Interactions		
Total debt # annual income	−0.096^***^	
	(0.025)	
Total debt # high-income group		−0.157^***^
		(0.040)
Control variables	Yes	Yes
Constant	7.923^***^	8.073^***^
	(0.230)	(0.236)

^+^*p* < 0.1, ^*^p < 0.05, ^**^p < 0.01, ^***^p < 0.001. Robust standard errors in parentheses. The coefficient estimates of the control variables and constants are not shown in the table.

Moreover, we used an FE model to examine the moderating effects of income, and the results support the findings above (see Supplementary Table S5). Therefore, these results supported Hypotheses 2.

#### 4.3.2 Social support

First, to test the moderating effects of monetary support, we added interactions between total debt and two measures of monetary support in predicting mental health (CESD-8 scores as the dependent variable). The results in Table 4 showed that only the interaction of total debt and monetary support from relatives could have a significantly negative impact on CESD-8 scores of adults (b = −0.044, *p* < 0.01), suggesting that debtors’ mental disorders could be mitigated by monetary support from relatives rather than friends. Specifically, when controlling for covariates, for every ten thousand yuan (RMB) increase in the value of monetary support from relatives, CESD-8 scores of indebted adults decreased by 0.044 points.

**Table 4:** OLS regressions on the moderating effects of monetary support.

Variables	Model 1	Model 2
	DV: CESD-8 scores	
Total debt (logarithm)	0.298^***^	0.303^***^
	(0.028)	(0.027)
Interactions		
Total debt # monetary support from relatives	−0.044^**^	
	(0.0145)	
Total debt # monetary support from friends		−0.077
		(0.231)
Control variables	Yes	Yes
Constant	7.726^***^	7.735^***^
	(0.240)	(0.238)

^+^*p* < 0.1, ^*^p < 0.05, ^**^p < 0.01, ^***^p < 0.001. Robust standard errors in parentheses. The coefficient estimates of the control variables and constants are not shown in the table.

Furthermore, we replaced the measures of monetary support with measures of emotional support (i.e., parent–child relationship quality and couple relationship quality) to test the buffering effects of emotional support. The results in Table 5 showed that all interaction terms of two measures of emotional support and total debt were not significant, indicating the weak roles of emotional support on mental disorders among indebted adults. Overall, the results partially supported Hypothesis 3a.

**Table 5:** OLS regressions on the moderating effects of emotional support.

Variables	Model 1	Model 2
	DV: CESD-8 scores	
Total debt (logarithm)	0.728^*^	0.462^**^
	(0.321)	(0.142)
Interactions		
Total debt # parent–child relationship quality	−0.096	
	(0.074)	
Total debt # couple relationship quality		−0.046
		(0.030)
Control variables	Yes	Yes
Constant	14.905^***^	13.139^***^
	(2.479)	(0.310)

^+^*p* < 0.1, ^*^p < 0.05, ^**^p < 0.01, ^***^p < 0.001. Robust standard errors in parentheses. The coefficient estimates of the control variables and constants are not shown in the table.

## 5 Conclusion and discussions

Given the rapid expansion of the credit market in emerging economies, especially in China, exploring whether indebtedness, as a money-related stressor, leads to mental disorders is becoming increasingly important. Based on a large sample of Chinese adults from CPFS (*n* = 41,274), we examined the relationship between indebtedness and mental disorders in the Chinese context and further explored the moderating roles of two types of coping resources (i.e., income and social support).

Although some studies conducted in developed countries have examined the association of indebtedness with mental health using the stress process model, they provided mixed evidence (7–12, 29). In line with the stress process paradigm, our results indicated that household debt had adverse effects on mental health among Chinese adults. This finding was convincing because we used other measures of core variables and reduced concerns that the results may be biased due to the endogeneity in baseline models and the sample selection bias. Thus, this study confirmed that indebtedness was a stressor of debtors’ mental disorders. The debt-mental disorders linkage can be attributed to the following social psychological mechanisms. On the one hand, indebted adults had to allocate current resources to repay debt or further borrow money to pay for necessities, which resulted in the deduction of disposable resources and limited their purchases of high-quality goods and services conducive to mental health (53). On the other hand, indebtedness has long been a sign of inability to restrain one’s own desire and thus was severely criticized in Chinese ideology (34). Therefore, individuals from indebted households may have feelings of shame by being socially discriminated against by others, which can serve as a source of mental disorders.

Additionally, the current study provided evidence for the link between indebtedness and mental health for the first time in the Chinese context. Indeed, household debt increased faster in emerging economies than in developed countries. Notably, China, as the second largest economy in the world, has the most rapid growth of household debt among emerging economies. Although existing studies conducted in China have indicated the adverse microeconomic effects of indebtedness at the household level, such as a decrease in income and a reduction in expenditure for survival (54, 55), it remains unclear whether household debt pertains to individuals’ mental health. To fill this research gap, this study investigated the mental health outcomes of indebtedness, suggesting the necessity of further research to examine the effects of household debt on individuals’ psychological development in emerging economies.

In line with the stress-buffering model, our results showed that income, as a critical personal resource, played a protective role against debtors’ mental disorders. Specifically, debtors who lived in high-income households had fewer mental disorders than those in low/middle-income households. This finding supported the anticipation of existing studies conducted in developed countries. On the one hand, it supported the expectation of Brown et al., i.e., the negative mental health outcomes of indebtedness could be alleviated by an increase in income (40). On the other hand, it was consistent with the findings of Hochman and Skopek, where high-income groups had greater life satisfaction than low/middle-income groups in times of financial crisis (56). Generally, income is tightly associated with borrowers’ abilities to repay debt. Consequently, high levels of income enable debtors to keep the balance between the usage of debt and disposable resources, which may produce positive material benefits and further protect mental health from indebtedness.

As an external coping resource, social support was selected as another moderator in this study following the stress-buffering paradigm. Specifically, the moderating effects of monetary and emotional support were examined in this study. Our results revealed that monetary support could moderate the linkage of indebtedness with mental disorders, but only monetary support from relatives played a significant moderating role. In China, relatives and friends have long been not only the main channels for borrowing money (57), but also the primary means of seeking help when individuals encounter economic hardship. However, with the rapid shrinkage of family size in China, the nuclear family has become the dominant family structure, which has weakened family members’ abilities to withstand external shocks (58). As a result, individuals have to rely more on their relatives to cope with numerous stressful life events. Thus, their physical and psychological development is more likely to be affected by support from relatives. As shown in Table 1, the mean value of economic help from respondents’ relatives was 5.5 times as much as that from friends. This descriptive exercise highlighted the potential differences in monetary support between the two sources, which offered the enlightenment that future research could further explore how monetary support from different sources produces different moderating effects.

Moreover, we found little evidence that emotional support, mainly provided by respondents’ spouses and children, had a buffering effect on the link between indebtedness and mental disorders in China. Our results were inconsistent with the findings of Yu and Liu, where emotional support could protect Chinese older adults’ mental health from stressful life events (18). This discrepancy could be attributed to the differences in the types of stressors. Specifically, indebtedness was a money-related stressor in the current study, but stressors in the cited research were associated with emotion (e.g., widowhood, divorce, and bereavement of children and relatives) or health (limited daily activities, poor self-rated health, functional disabilities, and hospitalization). This fact indicated that emotional support could buffer against higher stress triggered by economic hardship, such as indebtedness. It would be fruitful for future research to explore mechanisms involved in the invalid role of emotional support in debtors’ mental health.

This study had several limitations. First, given the differences in cultural settings, it was uncertain that the conclusions based on the Chinese data could be generalized to other emerging economies. Thus, cross-cultural research is needed to test the link between indebtedness and mental health in emerging economies. Second, even though this study used multiple methods to test the robustness of results, a carefully designed experiment is needed in future research concerning the causal direction from indebtedness to mental health. Finally, we had no information about borrowing motivation as well as the exact perception of economic pressure of respondents. Such information is conducive to establishing the causal relationship between indebtedness and mental health.

In sum, to the best of our knowledge, this was the first study to provide evidence on the association between indebtedness and mental health and the moderating effects of coping resources, including income and social support, within the stress-process and stress-buffering models in emerging economies. Therefore, our study contributed to the literature on the link between debt and mental health. On the one hand, the present study offered important policy implications for preventing the adverse effects of indebtedness. Specifically, our results indicated that indebtedness was an increasingly important stressor associated with mental disorders. Meanwhile, economic resources, such as personal income and external monetary support, were protective factors against mental disorders. These findings highlighted the need for building a support system that not only offers more opportunities to increase income by creating more jobs and perfecting economic systems, but also is combined with social networks based on the relationships of relatives.

## Data availability statement

Publicly available datasets were analyzed in this study. This data can be found here: http://www.isss.pku.edu.cn/cfps/.

## Author contributions

JL: Conceptualization, Data analysis, and Writing – original draft. YZ: Writing – review & editing. All authors contributed to the article and approved the submitted version.
