# Supplementary material for: Indebtedness and mental health in China: the moderating roles of income and social support
Source: Front Public Health. 2024 Jan 4;11:1279683. doi: 10.3389/fpubh.2023.1279683 (PMC10794583; doi:10.3389/fpubh.2023.1279683)
Supplement: Supplementary file 1 [file Table_1.DOCX]

TABLE S1. Fixed-effect regressions on the association of indebtedness with mental health.

| Variables | Model 1 | Model 2 | Model 3 |
| --- | --- | --- | --- |
|  | DV: CESD-8 scores | | |
| Any debt | 0.195^***^ |  |  |
|  | (0.041) |  |  |
| Total debt (logarithm) |  | 0.104^***^ |  |
|  |  | (0.018) |  |
| Total debt to assets |  |  | 0.126^***^ |
|  |  |  | (0.037) |
| Total assets (logarithm) | -0.085^***^ | -0.100^***^ | -0.072^**^ |
|  | (0.025) | (0.025) | (0.024) |
| Control variables | Yes | Yes | Yes |
| Constant | -0.100 | 0.010 | -0.087 |
|  | (1.841) | (1.840) | (1.841) |

^+^*p* < 0.1, ^*^*p* < 0.05, ^**^*p* < 0.01, ^***^*p* < 0.001. Robust standard errors in parentheses. The coefficient estimates of the control variables and constants are not shown in the table.

TABLE S2. Probit and OLS regressions on the association of the instrument variable with the dependent variables.

| Variables | Model 1 | Model 2 | Model 3 |
| --- | --- | --- | --- |
|  | DV: Any debt | DV: Total debt (logarithm) | DV: Total debt to assets |
| *TDCL* | 0.043^***^ | 0.025^***^ | 0.004^***^ |
|  | (0.005) | (0.006) | (0.000) |
| Control variables | Yes | Yes | Yes |
| Constant | -0.528^***^ | -0.150^*^ | 0.187^***^ |
|  | (0.103) | (0.074) | (0.026) |

^+^*p* < 0.1, ^*^*p* < 0.05, ^**^*p* < 0.01, ^***^*p* < 0.001. Robust standard errors in parentheses. The coefficient estimates of the control variables and constants are not shown in the table. *TDCL* represents the amount of total debt aggregated at the community level.

TABLE S3. 2SLS regression on the effect of indebtedness on mental health.

| Variables | Model 1 | Model 2 | Model 3 |
| --- | --- | --- | --- |
|  | DV: CESD-8 scores | | |
| Any debt | 1.273^***^ |  |  |
|  | (0.256) |  |  |
| Total debt (logarithm) |  | 0.287^***^ |  |
|  |  | (0.066) |  |
| Total debt to assets |  |  | 1.738^***^ |
|  |  |  | (0.361) |
| Control variables | Yes | Yes | Yes |
| Constant | -221.646^***^ | -218.552^***^ | -188.045^***^ |
|  | (14.156) | (14.577) | (17.888) |
| Durbin-Wu-Hausman test | chi2 (1) = 13.32 | chi2 (1) = 11.59 | chi2 (1) = 10.44 |
|  | Prob > chi2 =  0.001 | Prob > chi2 = 0.001 | Prob > chi2 = 0.001 |
| Wald F statistic | 710.929 | 2667.239 | 368.921 |

^+^*p* < 0.1, ^*^*p* < 0.05, ^**^*p* < 0.01, ^***^*p* < 0.001. Robust standard errors in parentheses. The coefficient estimates of the control variables and constants are not shown in the table.

TABLE S4. PSM on the effect of indebtedness on mental health.

| Estimation methods | DV: CESD-8 scores | | |
| --- | --- | --- | --- |
|  | ATT | Std. Err. | t-value |
| NM | 0.739^***^ | 0.053 | 13.82 |
| RM | 0.724^***^ | 0.041 | 17.74 |
| KM | 0.721^***^ | 0.041 | 17.74 |
| LM | 0.729^***^ | 0.053 | 13.62 |

^+^*p* < 0.1, ^*^*p* < 0.05, ^**^*p* < 0.01, ^***^*p* < 0.001. Robust standard errors in parentheses. The coefficient estimates of the control variables and constants are not shown in the table. This table reports average treatment effect on the treated (ATT) using nearest neighbor matching (NM), radius matching (RM), kernel matching (KM), and local linear matching (LM).

TABLE S5. Fixed-effect regressions on the moderating effect of income.

| Variables | Model 1 | Model 2 |
| --- | --- | --- |
|  | DV: CESD-8 scores | |
| Total debt (logarithm) | 0.157^**^ | 0.176^***^ |
|  | (0.048) | (0.049) |
| Interactions |  |  |
| Total debt # annual income | -0.023^***^ |  |
|  | (0.006) |  |
| Total debt # high-income group ^a^ |  | -0.050^***^ |
|  |  | (0.013) |
| Control variables | Yes | Yes |
| Constant | -0.038 | -0.057 |
|  | (1.841) | (1.842) |

^+^*p* < 0.1, ^*^*p* < 0.05, ^**^*p* < 0.01, ^***^*p* < 0.001. Robust standard errors in parentheses. The coefficient estimates of the control variables and constants are not shown in the table.
